# Editorials

**Published:** 1898-09

**Authors:** 


### EDITORIAL.

### AMERICAN VETERINARY MEDICAL ASSOCIATION.

On the thirty-fifth anniversary of the United States Veterinary Medical Association was born the American Veterinary Medical Association at Omaha. It enters upon its career in the height of manhood, at the zenith of National Association power. The history of the United States Veterinary Medical Association has been made and recorded, and with many trials and vicissitudes counted among its experiences, yet the Association under its new name owes all its acquired strength and power to its parent. Reared and fostered in the earliest history of veterinary science in our land, it has counted among its list of members the strongest and most progressive members of the profession. On its roll of membership may be found the names of those who have led in the rôle of advancement and progress in every aspect of our professional progress. In teaching, in higher professional attainment, in the field of surgery and medicine, in journalism, in the building up of American veterinary literature, her members have attained honor, fame, and renown, and these priceless records fall to the heirship of the new and broader Association. This history, these records of work well done, these achievementf of our profession in nation, State, and city, come to the Association of the future, charged with weighty responsibilities for us to carry and discharge with equal fidelity. Well it is that we can say and feel that in the All Americas now to make up our organization, the strength in numbers and in individual power never was greater and more united in the common purpose that keeps us together, of bettering our profession; and the future will, as surely as the passing of the noonday sun of to-morrow, write on its pages a record os greater advancement, progress, and power.

Let the veterinarians of the All Americas now add their strength and worth to the American Veterinary Medical Association, and veterinary sanitary science, surgery, medicine, education, journalism, and allied branches of our work will move forward with rapid strides and fittingly close the work of the nineteenth century.

---

Veterinarian C. D. McMurdo, of the army, sent a special report to the Association as to the true condition of army veterinarians.

Army legislation was dealt with forcibly, and the War Department was called upon for explanations of why our profession was so treated.

President Salmon's address was a masterly one, and a deep and lucid presentation of the advancement of comparative medical science.

Tuberculosis, Texas fever, hog-cholera, and swine-plague, were themes of the address treated of in a light of the greatest importance scientifically and commercially.

Committee on Intelligence and Education's report was presented by the Chairman, Dr. Leonard Pearson, and was replete with excellent thoughts and suggestions.

Not a State east of the Empire Commonwealth was represented at Omaha. For twenty-five years this was the centre of membership of the U. S. V. M. A., and in the territory of New England are still to be found a large percentage of the oldest members in years.

The Omaha meeting exceeded the highest expectations of any of those who were present in the splendid programme provided; and Omaha will be long remembered by those present as having covered herself with honor in the royal manner in which she entertained her guests.

---

The JOURNAL is glad to note the return of S. Brenton to the teaching staff of the Detroit College of Medicine, Veterinary Department. Associated with Dr. E. A. A. Grange and others, this school is sure to be strengthened in many ways and become a valuable factor in higher veterinary education.

